# Meta‐analysis of massively parallel reporter assays enables prediction of regulatory function across cell types

**DOI:** 10.1002/humu.23820

**Published:** 2019-06-18

**Authors:** Anat Kreimer, Zhongxia Yan, Nadav Ahituv, Nir Yosef

**Affiliations:** ^1^ Department of Electrical Engineering and Computer Sciences, Center for Computational Biology University of California Berkeley California; ^2^ Department of Bioengineering and Therapeutic Sciences University of California, San Francisco San Francisco California; ^3^ Ragon Institute of MGH MIT and Harvard Cambridge Massachusetts; ^4^ Chan Zuckerberg Biohub San Francisco California

**Keywords:** functional genomics, gene regulation, machine learning, massively parallel reporter assays, regulatory variation, SNVs

## Abstract

Deciphering the potential of noncoding loci to influence gene regulation has been the subject of intense research, with important implications in understanding genetic underpinnings of human diseases. Massively parallel reporter assays (MPRAs) can measure regulatory activity of thousands of DNA sequences and their variants in a single experiment. With increasing number of publically available MPRA data sets, one can now develop data‐driven models which, given a DNA sequence, predict its regulatory activity. Here, we performed a comprehensive meta‐analysis of several MPRA data sets in a variety of cellular contexts. We first applied an ensemble of methods to predict MPRA output in each context and observed that the most predictive features are consistent across data sets. We then demonstrate that predictive models trained in one cellular context can be used to predict MPRA output in another, with loss of accuracy attributed to cell‐type‐specific features. Finally, we show that our approach achieves top performance in the Fifth Critical Assessment of Genome Interpretation “Regulation Saturation” Challenge for predicting effects of single‐nucleotide variants. Overall, our analysis provides insights into how MPRA data can be leveraged to highlight functional regulatory regions throughout the genome and can guide effective design of future experiments by better prioritizing regions of interest.

## 1 INTRODUCTION

Massively parallel reporter assays (MPRA; Weingarten‐Gabbay & Segal, 2014) provide cost‐effective, high‐throughput activity screening of thousands of sequences and their variants for regulatory activity (Kheradpour et al., 2013; Melnikov et al., 2012; Mogno, Kwasnieski, & Cohen, 2013; Patwardhan et al., 2012; Sharon et al., 2012; Smith et al., 2013). In these assays, a library of putative regulatory elements is cloned and then transfected or infected into cells of interest. Each element is either associated with a unique barcode or can serve as a unique barcode itself (Arnold et al., 2013). The activity associated with each given regulatory element (i.e., MPRA output) is assessed by sequencing the transcribed barcodes and estimating the ratio between the transcribed RNA and the construct's DNA. As MPRA is still a nascent technology, the development of computational tools that take advantage of existing MPRA data sets could help improve future MPRA candidate sequence selection, enhance our ability to predict functional regulatory sequences, and increase our understanding of the regulatory code and how its alteration can lead to phenotypic consequences.

Previous work have used single MPRA data sets to better identify functional DNA sequences and then study the features that make a sequence regulatory active (Grossman et al., 2017; Lee et al., 2015; Sharon et al., 2012). For example, in the expression quantitative trait loci (eQTL) causal SNP challenge of the Fourth Critical Assessment of Genome Interpretation (CAGI4) community experiment, participants developed methods for predicting regulatory activity of candidate genomic regions and the effect of minor variants on their regulatory potential in MPRA (Beer, 2017; Kreimer et al., 2017; Zeng, Edwards, Guo, & Gifford, 2017). The main lessons learned from this community effort highlighted the effectiveness of ensembles of nonlinear methods, especially when used on features related to transcription factor (TF) binding and chromatin accessibility. Interestingly, epigenetic properties predicted from DNA sequence (Alipanahi, Delong, Weirauch, & Frey, 2015; Zeng, Hashimoto, Kang, & Gifford, 2016; J. Zhou & Troyanskaya, 2015) were shown to be more predictive features than experimentally measured epigenetic properties.

Although these efforts provided an important first step, each of them focused on a single MPRA data set in a specific cellular context. Critical questions, therefore, remain as to how generalizable the insights from MPRA experiments are to other data sets or other cellular contexts. Here, we present a first comprehensive analysis of several MPRA data sets collected by different labs and in various cellular systems; these data sets explore the effect of endogenous loci in several different cell‐types. We derive a large set of properties to characterize each putative regulatory region and compare the performance of different methods and features for predicting MPRA output. We show that MPRA activity is predictable and that prediction methods tend to perform consistently well when tested on different data sets, with better performance for nonlinear methods and favorable results when using an ensemble approach. Consistently, the predictive capacity of individual features is comparable across data sets, with TF binding and epigenetic properties being the top predictors.

We next turned to investigate the generalizability of our models across data sets, which allowed us to distinguish between determinants of MPRA activity that are dependent on the cellular context (e.g., protein milieu in the cell) versus ones that are intrinsic to the DNA sequence. Here, we demonstrate that predictive models trained in one cellular context can be used to predict the MPRA output in another with reduced prediction power and that, as expected, regions whose activity is cell‐type specific are harder to predict in this cross‐ data set setting. We also observe that gene expression of TFs is overall consistent with the predictive ability of their binding instances, with highly expressed TFs being generally more predictive of MPRA activity. When comparing pairs of data sets for TFs that are predictive of MPRA activity, we notice that in some cases, TFs with cell‐type‐specific functionality are better predictors in that cell‐type.

In addition, we wanted to evaluate the applicability of our predictive models in studying the function of naturally occurring mutations. We, therefore, tested the ability of our framework to detect the effects of small variants—single‐nucleotide variants (SNV) or short insertions or deletions (indels)—on MPRA activity and achieved similar accuracy to the state of the art methods (Zeng et al., 2017). Finally, we applied our approach to the Regulation Saturation challenge of the Fifth Critical Assessment of Genome Interpretation (CAGI5), and demonstrate that it achieves top performance in identifying functional effects of SNVs in supervised settings.

## 2 METHODS

### 2.1 MPRA data sets

We used five publicly available MPRA data sets and one unpublished data set. (a) *K562*—putative regulatory regions (Kwasnieski et al., 2014) selected from ENCODE‐based annotated regions in K562 cells (Encode‐Project‐Consortium, 2012; Ernst & Kellis, 2010; Hoffman et al., 2013). This set includes 600 regions annotated as enhancers, 600 as weak enhancers, 300 as repressed in K562 cell line, 600 enhancer predictions from the H1hESC cell line that are not annotated as weak enhancers or enhancers in K562 cells, and 1,136 negative controls—random sequences from each class above were chosen and scrambled while maintaining dinucleotide content. The regions range from 121 to 130 base pairs and were tested in episomal context in K562 cells. Data from all sequences were used to fit MPRAnalyze, although only the 1,500 regions annotated with the K562 cell line were used in the remaining analyses. (b) *LCL*‐eQTL—78,738 regions (Tewhey et al., 2016) that contain an eQTL in LCLs, 150 base pairs, tested in episomal context in LCL. (c) *HepG2‐eQTL*—the same set of elements (Tewhey et al., 2016) as above, tested in episomal context in HepG2 cell line instead of LCL. For both data sets 2 and 3, all of the 78,738 regions were used to fit MPRAnalyze, whereas 3,044 regions corresponding to the first test group in the CAGI4 challenge (Kreimer et al., 2017) were used for the remaining analyses. (d) *HepG2‐chr*—2,236 candidate liver enhancers (Fumitaka Inoue et al., 2017) and 102 positive and 102 negative control sequences. Each sequence is 171 base pairs and tested in chromosomal context. (e) *HepG2*‐*epi*— the same set of elements (Inoue et al., 2017) as above, tested in episomal context. For both data sets 4 and 5, all regions were used to fit MPRAnalyze and the 2,236 candidate enhancer regions were used for the remaining analyses. (f) *hESC*—2,464 putative enhancer regions (Inoue et al., 2018) and 200 negative controls. Each region is 171 base pairs and tested in chromosomal context in hESC cell line. All regions were used to fit MPRAnalyze, whereas only the 2,268 candidate enhancer regions were used for the remaining analyses.

### 2.2 Quantifying activity of regions using MPRAnalyze

For each data set, we obtain the RNA and DNA raw counts for each barcode. We obtain a quantitative measure of enhancer‐induced transcription using MPRAnalyze (Ashuach et al., 2019). MPRAnalyze assumes a linear relationship between the RNA and DNA counts, with the scaling parameter, denoted *ɑ*, as the transcription rate. The method uses a parametric graphical model to incorporate external covariates and dispersion estimates into quantifying *ɑ*.

The MPRAnalyze model assumes the DNA counts are Gamma‐distributed and that given the latent plasmid count, the RNA counts are Poisson‐distributed centered around the product of the plasmid count and *ɑ*. This results in a closed‐form negative‐binomial likelihood function for the RNA counts. External covariates such as barcode effect, batch effects and conditions of interest are then incorporated into the model by constructing a pair of nested generalized linear models: One using the DNA counts to estimate the latent plasmid counts, and the other using these latent plasmid counts along with the RNA raw counts to estimate *ɑ*.

Classification of active/inactive enhancers is done by using the fitted *ɑ* values. If a data set has control regions (*K562* and *hESC*), we first calculate a robust version of the standard score from the *ɑ* values by subtracting the median over the control regions and dividing by the median absolute deviation (MAD) of the control regions. If no control region exists for the data set, we perform the previous step with the median and MAD over all regions instead of just the control regions. We then compute the survival function for each standard score and apply the Benjamini–Hochberg (BH) correction. The active regions are then defined as regions with a false discovery rate (FDR) of less than 0.05.

### 2.3 Features

We assessed the correlation of 56 single features (Table S1) with MPRA activity.

(a) *#GC; #polyA, #polyT*—number of G/C in the sequence; length of longest polyA/T subsequence. (b) *#5‐mers*—number of distinct 5‐mers in the sequence. (c) *MGW, Roll, ProT, HelT*—DNA shape features (T. Zhou et al., 2013) quantifying minor groove width, roll, propeller twist, and helix twist. (d) *Conservation*—evolutionary conservation score of region as predicted by phastCons (Siepel et al., 2005). (e) *Closest Gene Expression*—expression (TPM) of the closest gene from RNA‐seq data in the corresponding cell‐type. (f) *Promoter, Exon, Intron, Distal* —binary features indicating whether the element intersects a promoter, exon, and intron. *Distal* is defined to be 1 if the element does not intersect with either promoter, exon, or intron annotations. (g) *#motifs, Motif Density*—number of significant DNA‐binding ENCODE motifs (Encode‐Project‐Consortium, 2012) from simple DNA‐binding motif scoring (Grant et al., 2011), maximum number of motifs within a 20 bp window in the sequence. (h) *#deepsea‐top, #deepbind‐top*—number of TFs quantifications above 90th percentile across all the regions predicted by *DeepSEA* / *DeepBind*. (a) *#tf‐high, #tf‐med, #tf‐low*—number of TFs that are bound above 90th percentile by *DeepBind* and rank in the top, middle, or bottom 100 (out of 515) for RNA‐seq TPM in the relevant cell‐type. Note that for both (h) and (i), we do not retrain the *DeepSEA* and *DeepBind* models with additional data, but instead use the pre‐trained models to score each MPRA sequence. (j) <*factor>[Cell] Mean, TFBS Shuffled Mean*—mean across subsets of *Experimental* features. <factor> can be *TFBS*, *DNase*, *CTCF*, *Ezh2*, *H2az*, *H3K4me1*, *H3K4me2*, *H3K4me3*, *H3K9ac*, *H3K9me1*, *H3K9me3*, *H3K27ac3*, *H3K27me3*, *H3K36me3*, *H3K79me2*, *H4K20me1*, *P300*. For these factors we take the mean of the binary overlaps over all corresponding [, cell‐type specific to the data set's cell‐type,] *Experimental* features. *TFBS Shuffled Mean* is the mean across *n non* cell‐type specific, randomly chosen *TFBS* features, where *n* is the number of features in *TFBS Cell Mean*.

### 2.4 Statistical tests

We examine the predictivity of features and accuracy of prediction models using several statistical tests. For regression task—for example, predicting quantitative activity—we applied several correlation measures (Pearson, Spearman, and Kendall) considering either the entire test data or regions at the top 25% of quantitative activity; we also applied another Spearman's correlation test after first binning quantitative activity by quintiles. We refer to these seven tests as the *regression tests*. For classification task—for example, predicting active or not active—we record the area under receiver operating characteristic curve (AUROC) and area under precision‐recall curve (AUPRC); we refer to these two tests as the *classification tests*. The significance of each *regression task* was evaluated by the respective statistical test *q*‐values, which are obtained from *p*‐values via the Benjamini–Hochberg correction. The significance of classification was evaluated by the *q‐*values of the Kolmogorov–Smirnov test on the predictions with positive ground truth labels.

### 2.5 Training and testing

We deterministically divide each data set into 10 sections; data sets with the same regions (*LCL‐eQTL* and *HepG2‐eQTL,* and *HepG2‐chr* and *HepG2‐epi*) are divided consistently. For the supervised case, we perform 10‐fold cross‐validation where each fold trains the model on nine training sections then evaluating on the remaining section. For the cross‐data set case, we perform 10‐fold cross‐validation where each fold trains the model on nine sections from the training data set, then evaluating on the corresponding remaining section in the last data set. We use the statistics from each fold to calculate the overall mean and standard deviation statistics.

When comparing cross‐data set learning performance between training on chromosomal MPRA data (*HepG2‐chr*) versus training on episomal MPRA data (*HepG2‐epi*), we observe that training on *HepG2‐chr* showed better results than *HepG2‐epi* 37 out of 40 times (comparing results across different statistical tests; Figure 3 and Table S4). Same regions were used for training and testing was done on the other four data sets.

### 2.6 Prediction models

We predict the quantitative activity from element features with four regression models and their ensemble. The four models are a linear regressor with ElasticNet regularization (Zou & Hastie, 2005) with 0.5 as the L1 and L2 regularization coefficients and a RandomForest regressor (Breiman, 2001), an ExtraTrees regressor (Geurts et al., 2006), and a GradientBoosting regressor (Zhu et al., 2009), each with 1,000 estimators. The ensemble method is implemented by taking the average prediction of all four regression models.

For the classification task, we use a RandomForest classifier (Breiman, 2001) and an ExtraTrees classifier (Geurts et al., 2006), each with 1,000 estimators, as well as their ensemble. The ensemble method averages the predicted probability from each classifier.

For both regression and classification, we define a shuffle model with the same composition as an ensemble model but shuffles the labels of the training set before training. This allows us to quantify the probability of producing our ensemble results by chance.

### 2.7 CAGI5 data processing

We predicted the variant impacts (positive, zero, negative) of 13,186 SNVs from five enhancers and nine promoters after training on 4,650 different SNVs from the same enhancers and promoters. For each SNV, we obtained the variant and wild‐type sequence, each of length 187–600, then featurized both variant and wild‐type with the 4,535 features that differ between variant and wild‐type: *Predicted epigenetic properties*, *DNA k‐mer frequencies*, *#GC*, *#polyA/T*, DNA shape features, and *conservation*; we collectively referred to these features as *Sequence features*.

For the *discrete challenge*, we concatenate the features from variant and wild‐type into a feature vector of size 9,070. We trained one multiclass classifier to predict the discrete impact for all promoter variants at once, and another classifier for the enhancer variants. For submitting to the *continuous challenge*, we used the same feature processing steps as in the *discrete challenge*, but trained regressors to predict the continuous impact.

We retrospectively discovered that concatenating wild‐type/variants features performs identically to taking their difference; we also retrospectively discovered that training a classifier for all promoters (enhancers) together performs better in classification, whereas training a separate regression per element performs better in regression. As such, for all analyses of the *continuous challenge* besides the original submission, we subtracted the corresponding wild‐type features from each of the 4,535 variant features and used this difference in features to predict the continuous impact via regression, separately for each element (enhancer/promoter).

### 2.8 CAGI5 models

Each multiclass classification model used in *discrete challenge* is an ensemble of five RandomForest classifiers and five ExtraTrees classifiers, trained to predict the discrete impact class (positive, zero, negative).

Each regression model used in the *continuous challenge* for predicting promoter variants (or predicting all promoter variants at once) is an ensemble of one RandomForest regressor, one ExtraTrees regressor, and one GradientBoosting regressor, whereas each regression models used for predicting enhancer variants (or predicting all enhancer variants at once) is an ensemble of five RandomForest regressors and five ExtraTrees regressors.

Each classifier or regressor consists of 1,000 estimators, and each estimator used in RandomForest or ExtreTrees models considers the square root of the total number of features when looking for best split.

## 3 RESULTS

We used five publicly available MPRA data sets and one unpublished data set collected at several labs using a range of experimental methodologies and cell‐types (Section 2). In all cases, the MPRA constructs were designed to test endogenous human DNA sequences, and not in‐silico designed synthetic sequences (Smith et al., 2013). Thus, each element tested in each data set is associated with a source genomic region. Each data set consists of approximately 2,000 sequences with length that varies between 121 and 171 base pairs (Section 2). Unless otherwise noted, the MPRA experiment was performed in an episomal context. The first data set (Kwasnieski, Fiore, Chaudhari, & Cohen, 2014), which we refer to as *K562* **,** consists of putative regulatory regions selected from ENCODE‐based annotated regions in *K562* cells (Encode‐Project‐Consortium, 2012; Ernst & Kellis, 2010; Hoffman et al., 2013). The second and third data sets, which we refer to as *LCL‐eQTL* and *HepG2‐eQTL* (Tewhey et al., 2016), consist of sequences that contain an eQTL in lymphoblastoid cell lines (LCLs). The same sequences were tested in LCL and HepG2 cells, thus forming the two data sets. Notably, the *LCL‐eQTL* data set was used as the primary source for the CAGI4 eQTL causal challenge (Kreimer et al., 2017). The fourth and fifth data sets (Inoue et al., 2017) include candidate liver enhancers, tested in either episomal or chromosomal context. We refer to these data sets as *HepG2‐epi* (for MPRA plasmids) and *HepG2‐chr* (for MPRA integrated in the genome). The sixth data set includes putative enhancer regions (Inoue, Kreimer, Ashuach, Ahituv, & Yosef, 2018) tested in chromosomal context in human embryonic stem cells (hESC). We refer to this data set as *hESC*.

Separately, for each data set, we applied MPRAnalyze (Ashuach, Fischer, Kreimer, Theis, & Yosef, 2019; Section 2; Figures S1–S3), a new tool for statistical analysis of MPRA data developed in our group, to obtain (a) MPRA output: A quantitative measure of enhancer‐induced transcription, computed as the ratio between the estimated abundances of transcribed RNA and the construct's DNA. These values are estimated by constructing a nested pair of generalized linear models that extract the ratio RNA/DNA as a measure of activity while controlling for various confounding factors, and (b) a binary label that identifies active/inactive enhancers, namely enhancers whose activity significantly deviates from that of the negative controls (median‐based *z*‐score; FDR < 0.05).

The CAGI5 Regulation Saturation challenge, also titled “Predicting individual non‐coding variant effects in disease associated promoter and enhancer elements,” experimentally assessed the effects of 17,500 SNVs in 14 regulatory elements that are associated with human disease. Specifically, nine promoters (F9, GP1BB, HBB, HBG, HNF4A, LDLR, MSMB, PKLR, and TERT) and five enhancers (IRF4, IRF6, MYC, SORT1, and ZFAND3) of lengths 187–600 base pairs (bps) were tested with saturation mutagenesis for MPRA activity in a relevant cell‐type (HepG2, HEL 92.1.7, HEK293T, K562, GBM, SK‐MEL‐28, HaCaT, and MIN6; Kircher et al., 2018).

### 3.1 Predictive features for MPRA activity are consistent across data sets

We first defined a set of features that characterize each MPRA sequence and inspected each feature individually (Section 2; see Table S1 for a complete description of all features). Overall, we examined 56 features that can be divided into four categories (similarly to Kreimer et al. (2017)). (a) *Experimentally measured epigenetic properties*: To define these, we mapped each assayed region to its corresponding position in the reference human genome, and then queried this position against tracks of epigenetic properties from ENCODE (Encode‐Project‐Consortium, 2012). These properties were measured in multiple cell lines and include the overall number of observed TF binding sites (TFBS), histone marks, binding by chromatin structure‐associated proteins (e.g., P300), chromatin accessibility (primarily by identifying DNase‐hypersensitivity sites; henceforth abbreviated as DHS), and DNA‐ methylation. For all these features we either aggregate over all available cell‐types, or restrict the analysis to the same cell‐type in which the MPRA was conducted. (b) *Predicted epigenetic properties*: This set of features covers similar properties as the experimentally derived ones (e.g., TFBS or histone marks). However, instead of being directly measured, the properties are inferred based on the DNA sequence of the respective MPRA construct, using models trained on experimental data (e.g., protein‐binding microarrays for TFBS [Newburger & Bulyk, 2009] or ChIP‐seq for histone marks [Encode‐Project‐Consortium, 2012]). We use three models for this purpose: scoring of protein‐DNA‐binding motifs (Grant, Bailey, & Noble, 2011), the more recent supervised methods *DeepBind* (Alipanahi et al., 2015), and *DeepSEA* (J. Zhou & Troyanskaya, 2015). In all three cases (motif scoring, *DeepBind*, and *DeepSEA*), we do not retrain the models with additional data, but only use the pre‐trained models to score each MPRA sequence. Another feature included here is *Motif Density*—defined as the maximum number of protein‐DNA‐binding motifs within a 20 bp window in the MPRA sequence. (c) DNA *k‐mer frequencies* using *k* = 5. And (d) *Additional locus specific features*: Here we used the number of G/C in the sequence (*#GC*) as well as the length of longest polyA/T subsequence (*#polyA/T*). We also used DNA shape features (T. Zhou et al., 2013) quantifying minor groove width, roll, propeller twist, and helix twist (*MGW, Roll, ProT, and HelT* respectively). Additional features in this category include: *Conservation*—evolutionary conservation score of region as predicted by phastCons (Siepel et al., 2005). *Closest Gene Expression*—expression (TPM) of the closest gene from RNA‐seq data in the corresponding cell‐type. *Promoter, Exon, Intron, Distal*—binary features indicating the respective location in the endogenous genome.

**Figure 1:**
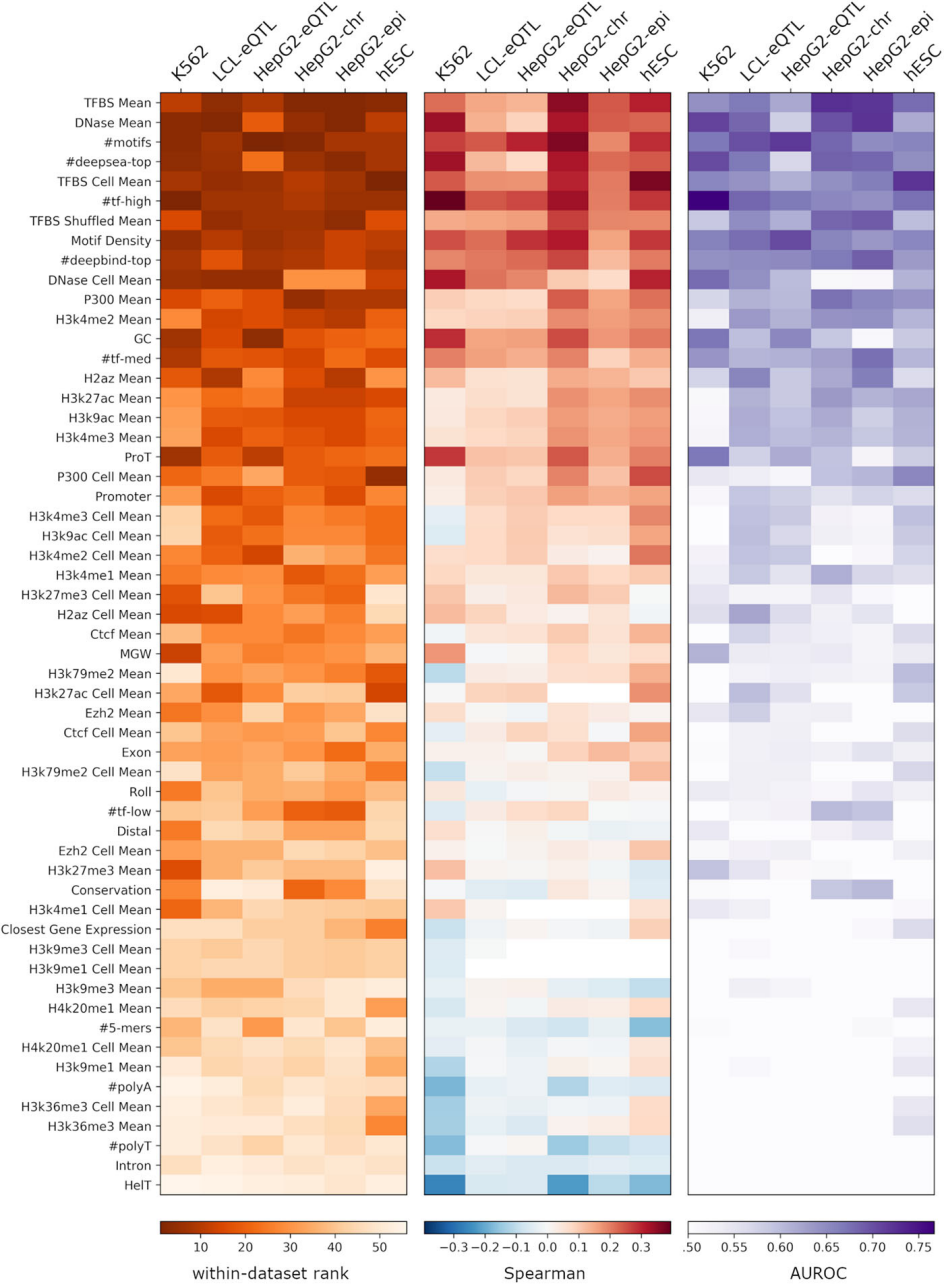
Individual feature correlation with massively parallel reporter assay output. The within‐data set ranking is calculated by first ranking each feature by each test, then taking the median of the regression and classification test rankings. The comprehensive ranking is the median across all the data set rankings. The heatmaps are ordered according to the comprehensive ranking and colored according to (a) the within‐data set rank, (b) the Spearman correlation coefficient for regression task, and (c) the area under receiver operating characteristic curve value for classification task

We use these 56 features (Section 2; Table S1) individually in two ways: (a) we test how well each feature correlates with the quantitative MPRA output of each data set using seven *regression tests* (Section 2) and (b) we test how well each feature discriminates between active and inactive regions using two *classification tests* (Section 2). We rank each feature for each of the nine tests and then take the median of these ranks to obtain a data set‐specific feature ranking. We then take the median across all data set‐specific ranking to obtain a global ranking of the features and sort them according to their global rank (Figure 1). Notably, the different statistical tests are largely consistent with the global rank (Figure 1 and Table S1), supporting its robustness. This global rank highlights chromatin accessibility (*DNase Mean*) and the number of TF binding sites (*TFBS Mean*) as the most predictive features for MPRA activity across all data sets. To gauge the robustness of our results, we repeated the above feature correlation experiments 100 times, each time sampling 80% of the loci in the data, and report the mean and standard deviation of the resulting accuracy (Table S1).

To further explore cell‐type specificity in the context of TF binding, we stratified the TFs into three groups according to their expression level in the cell‐type of interest (low/intermediate/high) and sum over the number of binding sites in each group. Although these three features *#tf‐high, #tf‐med, #tf‐low* had a strong correlation (especially *#tf‐high*) with MPRA activity (Figure 1), they are still less predictive than *TFBS Mean* (the simple mean across all TFBS‐related features). Consistently, we found several cell‐type agnostic features such as *GC* content and *#motifs* that are predictive of MPRA activity as well (Figures 1, S4, and S5).

Furthermore, we found that limiting the set of TFs in a manner specific to the cell‐type under investigation (e.g., for the *K562* data set, *TFBS Cell Mean* only considers TF ChIP‐seq experiments conducted in K562 cells) does not improve accuracy (Table S1), compared with taking all available data regardless of cell‐type of origin (*TFBS Mean*). This observation is consistent with previous work on enhancer annotation, showing that integration of diverse data sets from different cellular contexts improves developmental enhancer prediction over approaches based on single context data (Erwin et al., 2014). As additional control, we randomly subsampled *N* (the number of TFs used to calculate *TFBS Cell Mean*) ChIP‐seq experiments that were conducted in a cell‐type different from the one used for MPRA, and computed the mean number of binding sites. Consistent with the results above, we found that the predictive capacity of this random set of TFs‐binding scores (considering 100 randomly selected sets for each of our six data sets; denoted *TFBS Shuffled Mean*) is not lower than that of ChIP‐seq experiments conducted in cell‐type in which MPRA was conducted (empirical *p *> 0.25).

The CAGI5 saturation mutagenesis data set consists of a small number of endogenous genomic regions, selected in large under the assumption that they play important regulatory role in the respective cellular context. Indeed, these regions show significant amount of MPRA activity when tested in their corresponding cell‐types (Kircher et al., 2018). As such, one would expect that these regions might exhibit higher than usual values in the aforementioned set of features. To test this, we featurized these 14 regions as above and compared the signal of each feature to the distribution of the same features across regions in *LCL‐eQTL* data set and reported the percentile per feature (Figure S6). We chose *LCL‐eQTL* as the background distribution since regions tested in this experiment were selected based on harboring an eQTL variant, which provides a potentially weaker indication for regulatory activity (Tewhey et al., 2016; as opposed to experimental designs that selects for regions that are enriched with regulatory element marks—e.g., H3K27ac peaks). As CAGI5 regions have varying lengths, we focused our analysis on 150 bp in the center of each region (corresponding to sequence lengths of 150 bp in *LCL‐eQTL*). As expected, for the most predictive features, we observe a higher than median signal in CAGI5 tested regions comparing with LCL‐eQTL regions (Figure S6).

### 3.2 Predictive models of MPRA activity are similar across data sets

Next, we turned to the construction of supervised models that are trained to predict the MPRA output either as a quantitative measure of enhancer activity (i.e., regression task) or as a binary label that distinguishes between active and inactive enhancers (i.e., classification task). To this end, we considered a collection of regression models (*Elastic Net* [Zou & Hastie, 2005], *Random Forest* [Breiman, 2001], *Extra Trees* [Geurts, Damien, & Louis, 2006], *Gradient Boosting* [Zhu, Zou, Rosset, & Hastie, 2009], and *ensemble*) and classification models (*Random Forest* [Breiman, 2001], *Extra Trees* [Geurts et al., 2006], *ensemble*), which we applied separately for each data set. We trained these models using a set of features that extends the one investigated in Figure 1 **,** with the following categories: (a) *Experimentally measured epigenetic properties*—1,095 binary features based on ENCODE data (Encode‐Project‐Consortium, 2012). These features indicate whether the genomic region overlaps with experimentally measured tracks of: TFBS from ChIP‐seq experiments, histone modifications, and DNase‐hypersensitivity sites across different cell‐types (Table S1). (b) *Predicted epigenetic properties—*This set consists of three sources: (a) *DeepBind*—515 features, each indicating a binding score of a certain TF, predicted by a sequence‐based neural network model trained on protein‐binding microarrays (Alipanahi et al., 2015). (b) *DeepSEA*—919 binary features, indicating predictions of various events related to chromatin structure, namely TF binding, DNA accessibility, and histone modifications. These events were predicted by a sequence‐based neural network model trained on ENCODE data (J. Zhou & Troyanskaya, 2015). (c) *Motifs*—2,065 binary features indicating motif hits (Encode‐Project‐Consortium, 2012; Grant et al., 2011; Kheradpour & Kellis, 2014). (c) DNA *k‐mer frequencies*—1,024 binary features, indicating the presence or absence of all possible nucleotide 5‐mers. (d) *Additional locus specific features* as in Figure 1 (Table S1).

**Figure 2:**
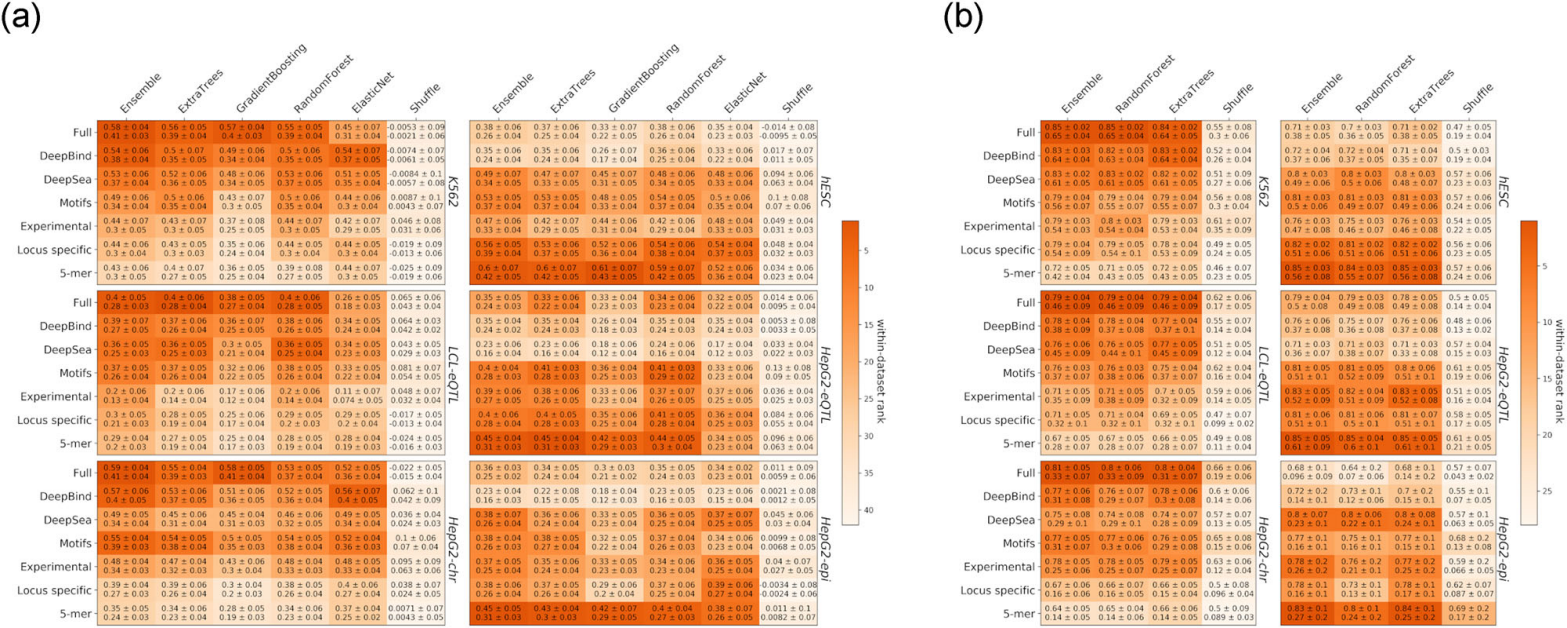
Performance of (a) regression models and (b) classification models with different feature combinations. The within‐data set ranking is calculated for each cell by taking the median of the rankings for all the (a) regression or (b) classification tests within a data set. Each heatmap is colored according to the within‐data set rankings. The statistics are mean ± standard deviation for (a) Spearman and Kendall tests or (b) area under receiver operating characteristic curve and area under precision‐recall curve tests

We evaluate the accuracy of prediction in each combination of data set × prediction method × feature category using 10‐fold cross‐validation. We report the mean and standard deviation of the resulting scores (Figure 2). Importantly, we do not use our evaluation of individual features in Figure 1 during model training (e.g., for feature selection), thus avoiding circularity.

Reassuringly, the accuracies of our top model for predicting MPRA activity on the *LCL‐eQTL* data set (regression and classification: 0.4 Spearman's correlation and 0.79 AUROC, respectively) matched that of the top ranking group in the CAGI4 challenge (0.34 Spearman's correlation and 0.8 AUROC; Zeng et al., 2017). Consistent with our results for single features, we observe an overall agreement in our results across data sets, both in terms of the relative performance of each algorithm, and in terms of the importance of each feature category. Specifically, we observe that nonlinear methods perform better (e.g., cf. elastic net to random forest) and that an ensemble approach (aggregating over all classifiers or regression methods) tends to have the highest performance (Figure 2 and Table S2). Among the feature categories, the predicted TF binding properties according to *DeepBind* are top performers, and the union of all feature categories generally yields the best performance, indicating that even with a large feature set the various models still do not over‐fit. To further test this, we trained our models on shuffled labels (Section 2), and observed that the performance significantly decreases in all cases, including the more complex ensemble model that uses the complete feature set.

Another result consistent with the ones observed with single features regards the importance of cell‐type specificity, where we again noticed that limiting the epigenetic features to be cell‐type specific does not increase accuracy (Figure S7 and Table S3). Finally, it is interesting to note that the accuracy achieved with a chromosomal MPRA library in HepG2 cells (*HepG2‐chr*) tends to be slightly higher than the one obtained with an episomal library (*HepG2‐epi*; regression: 0.59 vs. 0.45 Spearman correlation and 0.41 versus 0.31 Kendal correlation; Figure 2). These results are consistent with a recent comparison between these two experimental approaches (Inoue et al., 2017) that found chromosomal MPRA to be more reproducible, have higher correlation with epigenetic marks and work in variety of cell‐types that are harder to transfect (e.g., hESCs); however, more data sets are required to substantiate this finding.

### 3.3 Transferring knowledge between cell‐types

Using existing MPRA data to build models that can be applied across different cellular backgrounds and for genome‐wide predictions of regulatory elements can be useful for prioritizing functional regulatory regions, which can guide the design of new MPRA panels and used for analysis purposes. To evaluate how well our models generalize to a new cellular context where MPRA data is not available, we tested the extent to which models trained in each data set can be used to predict the outcome in the remaining data sets. Based on the results in Figure 2, we take the *Full* set of features (i.e., all feature categories) and use the *ensemble* model for both the regression and classification tasks. We avoid training on any genomic region from one data set (e.g., *LCL‐eQTL*) that is already in the test set from another data set (e.g., *HepG2‐eQTL*).

**Figure 3:**
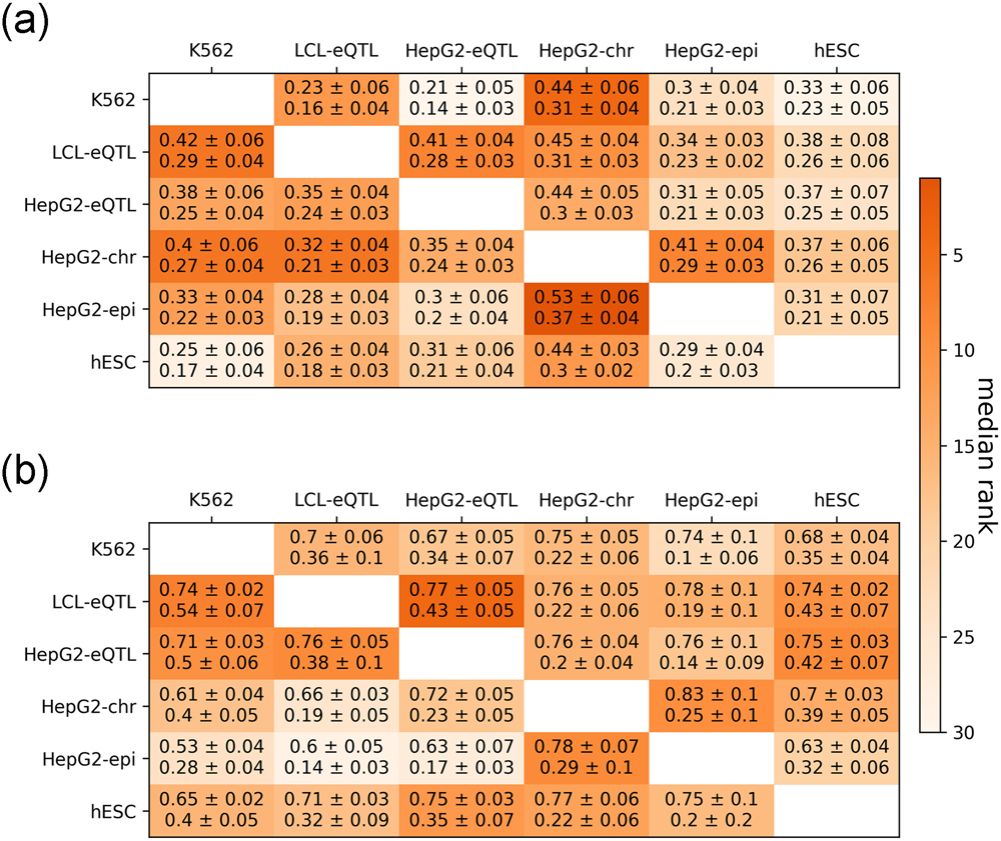
Performance of cross‐data set learning for (a) regression task and (b) classification task between cell‐types. All cross‐ data set learning models are ensemble models with full features. Each cell is colored according to the median over the ranks of all (a) regression tests or (b) classification tests. The statistics are mean ± standard deviation of (a) Spearman and Kendall tests or (b) area under receiver operating characteristic curve and area under precision‐recall curve tests

We observe that performance in the regression task is reduced in this cross‐data set setting compared with the supervised setting. For example, for the *K562* regression task, the best model trained on *K562* data achieves a cross‐validation Spearman of 0.58 (Figure 2 and Table S2), whereas the best models trained on *LCL‐eQTL*, *HepG2‐eQTL*, *HepG2‐chr*, *HepG2‐epi*, *hESC* data set only achieve Spearman's correlations of 0.23, 0.21, 0.44, 0.3, 0.33, respectively (Figure 3 and Table S4). However, performance in the classification task is generally robust. For example, for the *K562* classification task, the best model trained on *K562* data achieves an AUROC of 0.85 (Figure 2), whereas the best models trained on *LCL‐eQTL*, *HepG2‐eQTL*, *HepG2‐chr*, *HepG2‐epi*, *hESC* achieve AUROCs of 0.7, 0.67, 0.75, 0.74, 0.68, respectively (Figure 3 and Table S4). These results suggest that MPRA data in one cellular context can be leveraged to distinguish between regions of regulatory importance in another.

**Figure 4:**
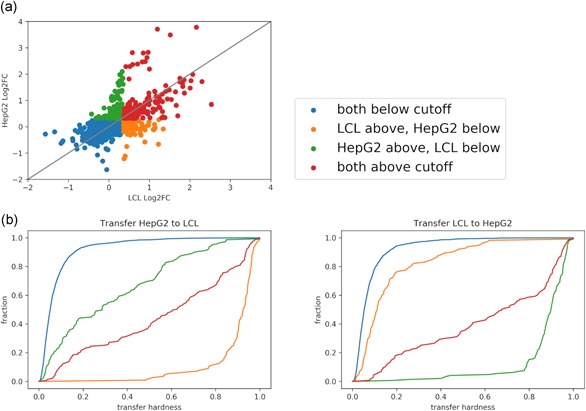
(a) *LCL‐eQTL* versus *HepG2‐eQTL* MPRA activity by log2 *ɑ* values. The points are colored according to activity in each of the data sets (active/inactive is defined as above/below 1.5 cutoff, respectively). (b) We define hardness as the rank‐normalized absolute difference between the ground truth binary activity label (0 or 1) and predicted probability. The cumulative distribution function of the hardness for each of the four activity groups when training the ensemble, full feature cross‐data set model on *HepG2‐eQTL* (Left subfigure) and *LCL‐eQTL* (Right subfigure), and testing on *LCL‐eQTL* and *HepG2‐eQTL*, respectively

We hypothesized that genomic regions that are uniquely active in a certain cell‐type would be harder to predict in a cross‐data set setting. To explore this, we took advantage of the *LCL‐eQTL* and *HepG2‐eQTL* data sets, which include the same set of genomic regions. We first examined the distribution of three region categories in these two data sets (Figure 4a): common regions (i.e., active regions in both data sets), cell‐type‐specific regions (i.e. regions active in one of the data sets), and inactive regions (i.e., regions not active in both data sets). We then examined prediction performance for each of the region categories (Figure 4b) in cross‐data set analysis where we apply the classifier built on one data set to annotate regions in the other data set as active or not. To assess this, we defined the “hardness” of the region based on the difference between the predicted score (in range [0, 1]) and the class label (1 for active and 0 for not‐active region). Reassuringly, we observe that cell‐type‐specific regions are harder to predict in cross‐data set learning (Figure 4b). These results suggest that while the MPRA signal can be predicted to some extent using cell‐type agnostic components, it also depends on cell‐type‐specific ones. Interestingly, and consistent with our cross‐validation (i.e., per‐ data set) analysis, we observe that the cross‐data set accuracy achieved with models trained on chromosomal MPRA library (*HepG2‐chr*) is higher (0.33, 0.28, 0.3, 0.31 Spearman's correlation and 0.53, 0.6, 0.63, 0.63 AUC for K562, *LCL‐eQTL*, *HepG2‐eQTL*, *hESC* respectively) than the one obtained with an episomal library (*HepG2‐epi*; 0.4, 0.32, 0.35, 0.37 Spearman's correlation and 0.61, 0.66, 0.72, 0.7 AUC for K562, *LCL‐eQTL*, *HepG2‐eQTL*, *hESC*, respectively; Section 2; Figure 3 and Table S4).

Finally, we wanted to examine the predicted MPRA signal of the 14 endogenous regions included in the CAGI5 data set, which were shown to induce transcription by MPRA when tested in their corresponding cell‐types (Kircher et al., 2018). To this end, we trained a regression model on *LCL‐eQTL* regions with the full set of features and used it to predict the MPRA activity of CAGI5 regions. As the sequence lengths in the *LCL‐eQTL* were 150 base pairs, we refeaturized the center 150 base pairs of the CAGI5 regions. We find that when comparing the predicted MPRA activity of CAGI5 regions with the distribution of *LCL‐eQTL* regions activity, most CAGI5 regions were in the >90th percentile (Figure S4). This is consistent with our expectation that the CAGI5 regions should be recognized as having a significant transcriptional activity.

### 3.4 Contributions of individual TFs to the accuracy of predicting MPRA outcome

**Figure 5:**
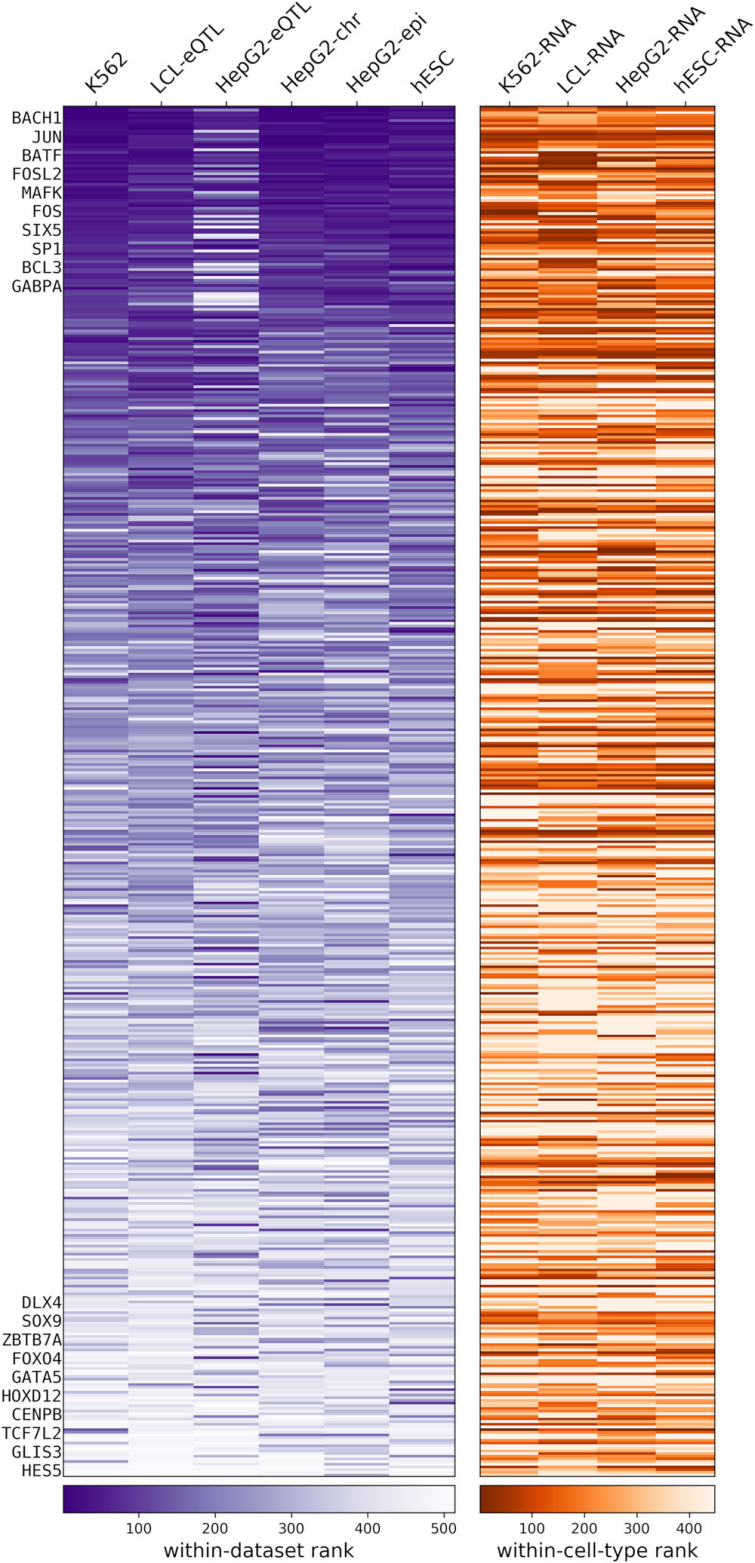
Contribution of individual DeepBind transcription factor (TF) binding for predicting regulatory activity of massively parallel reporter assay constructs. The within‐data set ranking is calculated by taking the per feature median rank across all classification and regression tests. The comprehensive ranking is the per feature median overall within‐data set rankings. TFs are sorted from best (smallest) to worst comprehensive rank. (Left) Heatmap of the within‐data set rankings. (Right) The per TF ranking of its messenger RNA levels measured by RNA‐seq in each of the four cell lines. Names of the common top/bottom 10 factors are indicated on the left

We wanted to explore which factors in different cells drive the activity of regulatory regions, and hypothesized that the protein milieu in the cell might act as one. To this end, we examined the contribution of individual TFs to MPRA activity. We recorded the correlation between each TF binding signal (*DeepBind* prediction) and the activity of each MPRA region (Alipanahi et al., 2015). Similarly to our analysis in Figure 1, we then ranked the TFs based on their predictive ability across data sets, thus revealing several TFs whose binding is generally informative of regulatory activity of MPRA constructs in all cellular contexts in this study (Figures 5, S8, and S9 and Table S5). For instance, two TF families with a data set‐wide high predictive capacity, that is also supported by experimentally‐evaluated binding from ChIP‐seq ( Figure S9 and Table S5) sites are *JUN* and *FOS*. Proteins of the *FOS* family dimerize with proteins of the *JUN* family, thereby forming the TF complex *AP‐1*, which has been implicated in a wide range of cellular processes, including cell growth, differentiation, and apoptosis across different cell‐types (Ameyar, Wisniewska, & Weitzman, 2003). More generally, we find that TFs whose binding is commonly predictive of MPRA activity across data sets are also highly expressed across all the three cell‐types, as indicated by RNA‐seq data (Figure 5). Indeed, the gene expression of TFs is overall consistent with their predictive capacity, whereby more predictive factors have overall higher expression as measured by RNA‐seq (Encode‐Project‐Consortium, 2012; Figure 5—right four columns) across all cell‐types (the Wilcoxon rank sum test of top vs. bottom 50 factors: *p*‐value of 3.9e−6, 1.36e−5, 8.7e−4, and 8.0e−4 for K562, LCL, HepG2, and H1hESC, respectively).

### 3.5 Exploring common and distinct TF binding between data sets

**Figure 6:**
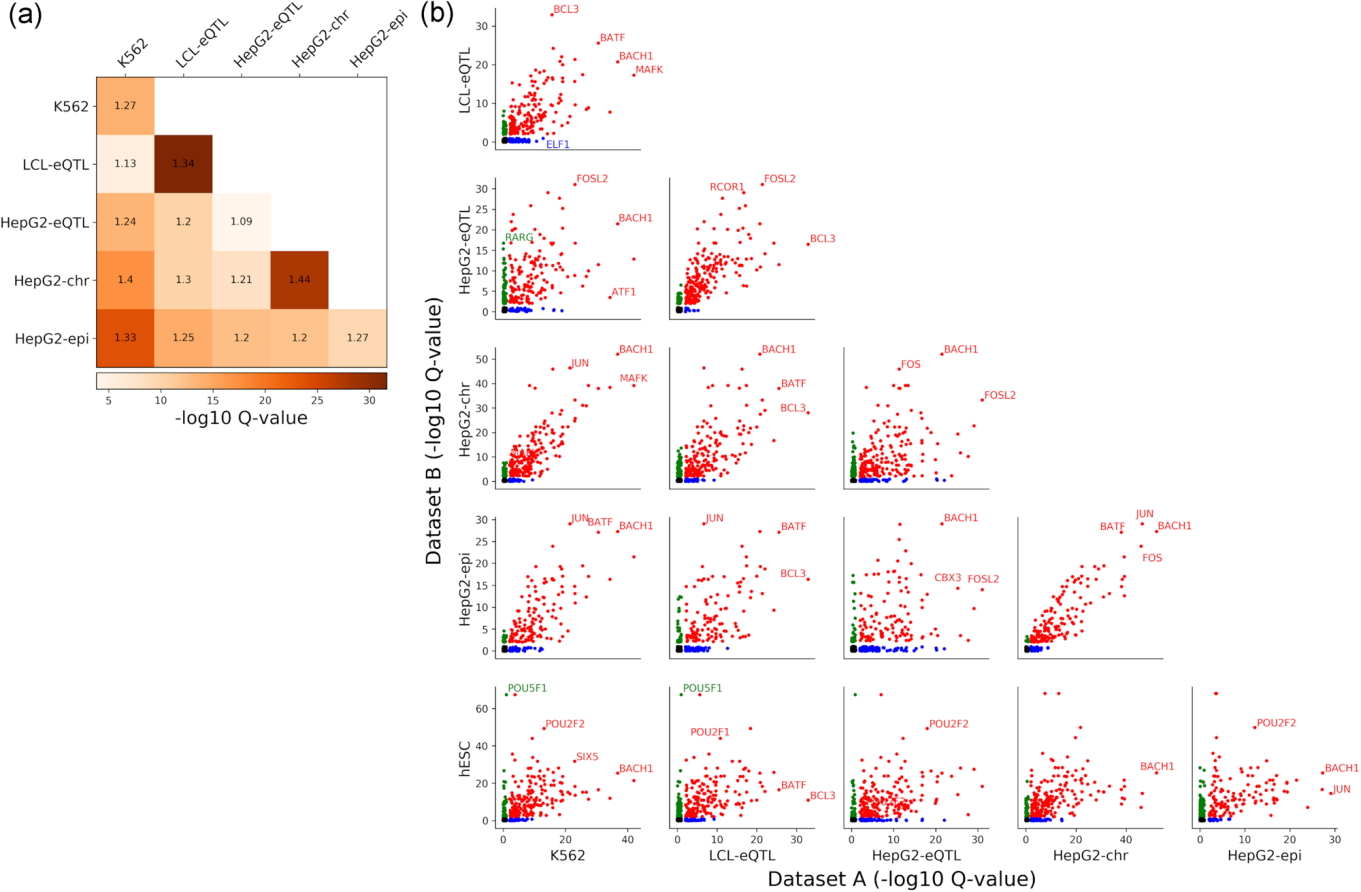
Similarities and differences in transcription factors (TFs) whose binding is predictive of Massively parallel reporter assay (MPRA) activity between data sets. For each data set, we define a set of *predictive TFs*, as the set of bound TFs (predicted by *DeepBind*) that is significantly correlated with MPRA output (Spearman's *q* < 0.05). (a) Similarity of *predictive TFs* between data sets. For each pair of data sets, a hypergeometric test is performed on the sets of *predictive TFs* of both data sets, resulting in a *q*‐value indicating the likelihood of the overlap occurring by chance (color scale). We also calculate the enrichment ratios of *predictive TFs* for each pair of data sets (cell text). (b) Differences in *predictive TFs* between data sets. For each data set, we only plot high confidence *predictive TFs* (i.e., significant TFs) that have Spearman's *q*‐values of less than 0.01, and non*predictive TFs* (i.e., nonsignificant TFs) have *q*‐values of greater than 0.1

We next proceeded to explore TFs whose binding is predictive of MPRA activity only in specific cell‐types. To this end, we defined, for each data set, a set of *predictive TFs*, as the set of bound TFs (predicted by *DeepBind*) that is significantly correlated with MPRA output (Spearman's FDR corrected *p *< 0.05). We then compare across pairs of data sets to determine if there is significant overlap in *predictive TFs*. To this end, for each pair of data sets, we calculated the fold enrichment of the overlap between the predictive TF set, and evaluated the significance of this overlap using a hypergeometric *p*‐value (Figure 6a). Overall, we see that there is significant overlap across every pair of data sets. Interestingly, the similarity between data sets seem to be dominated by the similarity between the MPRA sequences and less so by the similarity in cellular context. Specifically, the *HepG2‐chr* and *HepG2‐epi* pair and *LCL‐eQTL* and *HepG2‐eQTL* pair had the strongest overlap, with higher similarity between experimental versions tested in the same cell‐type (HepG2‐chr/HepG2‐epi) than same elements tested in different cell‐types (LCL‐eQTL/HepG2‐eQTL), suggesting that the same genomic regions tested in different conditions have correlated signals in MPRA. However, this result may depend on the specific sequences studied, and further data needs to be collected to substantiate it.

We further examine the *predictive TFs* that differ between pairs of data sets (Figure 6b and Table S6), and provide a list of top *predictive TFs* in at least one data set. In some cases, we find proteins whose function is related to the cell‐type under investigation. For instance, when comparing the two data sets with the lowest *similarity score* for *predictive TFs*, *K562* to *HepG2‐eQTL*, we find that *RARG* (a retinoic acid receptor which belongs to the nuclear hormone receptor family and is associated with liver risk phenotype [Roberts et al., 2010]) is predictive in *HepG2‐eQTL* but not *K562*. When comparing *K562* to *LCL‐eQTL*, we observed that the genes in the *ETS* family (*ELF1*, *ELF5*, *ELF3*, *ETV6*, *ELK3*) are predictive only in *K562*. These genes are known to be expressed in hematopoietic tissues and cell lines, and play a role in hematopoietic cell development (Clausen et al., 1997). When comparing *hESC* to the other data sets, we observe a known pluripotent factor‐ *POU5F1* (Boyer et al., 2005) to be predictive only in *hESC* for most of the comparisons (Table S6).

Overall, these results support the notion that both sequence‐intrinsic (i.e. derived solely from the sequence tested in MPRA) and cell‐type‐specific (i.e., derived from the cell‐type where MPRA was tested) properties are determining MPRA activity. We also find that the cell‐type‐specific component may be captured by the activity of TFs whose function is associated with the cell‐type under investigation.

### 3.6 Studying the effects of small genetic variants on MPRA output in CAGI5

MPRA can be used to study the transcriptional effects of small variants that commonly occur in regulatory regions, namely SNVs and small indels (Tewhey et al., 2016). We wanted to examine if we can predict these effects using our framework. To test this, we used the data from the CAGI5 challenge, which consisted of saturation mutagenesis analysis of 14 regions, overall testing the effects of 17,500 SNVs. The training data consisted of 25% of the saturation mutagenesis results in each of the 14 regions.

The participants in the CAGI5 challenge were asked to use the training data to predict the impact of SNVs on the MPRA signal in the held‐ out parts of the experiment. The challenge was divided in two. In the *discrete challenge part,* the goal was to predict the category of impact (negative effect, no effect, positive effect). The CAGI5 evaluators later (post submission) requested that the top performing groups each provide a prediction of the continuous impact of each SNV (namely, fold change of the MPRA signal); we refer to this as the *continuous challenge* (Shigaki et al., 2019).

For both the *discrete* and the continuous challenge, we featurized the wild‐type and variant sequences with all features that depend on the raw sequence, including *DeepSEA*, *DeepBind*, *DNA kmer‐frequencies,* and so forth (Section 2)—we call this collection of features the *Sequence features*; we excluded the features that depend on the genome coordinate, such as *Experimental* features, because those are the same for all variants of the same element. The inputs to our prediction problem are therefore pairs of sequences—a wild‐type feature and a mutated feature. To train a predictive model, we examined two ways to featurize each pair—either concatenate the feature vectors of the wild‐type and alternative allele, or subtract the values of the alternative allele from those of the wild‐type allele. We found that concatenation versus subtraction made very little difference in accuracy (data not shown) and thus focus our discussion on the latter.

For the *discrete challenge*, we trained a single multiclass (−1, 0, +1; denoting negative effect, no effect, and positive effect respectively) classification model to predict the discretized impacts of genomic variation. We trained a separate model for the effects of variants in promoter sequences and in gene‐ distal sequences (Section 2). The assessors of this challenge (Shigaki et al., 2019) evaluated accuracy using Pearson correlation of the predicted labels (−1, 0, 1) with the continuous MPRA expression impact scores. They also calculated the AUROC treating this as a discretized classification task (e.g., 1 vs. [0 and −1]).

Our analysis yielded the highest average accuracy across all the elements. Specifically, (0.318 Pearson and 0.249 Spearman; correlations are between the predicted scores and the −1, 0, 1 labels), as well as competitive average AUROCs (0.762 for positive vs. negative, 0.706 for positive vs. rest, 0.776 for negative vs. rest; Table S7).

For the *continuous challenge*, we used the aggregated features and trained one regression model to predict the continuous impacts of all promoter variants and one regression model to predict the impacts of all enhancer variants (Section 2). Across all the submissions, our continuous impact submission tied for the best average Pearson's correlation across all the elements (0.451 for ours vs. 0.452 for submission G3/cont1), and achieve best Pearson's correlation scores in 10 of the 14 elements (Table 1).

**Table 1:** Performance comparison for the CAGI5 Regulation Saturation *continuous challenge* for the nine promoters and five enhancers, sorted by average Pearson's correlation

Submission/regulatory element	G3/cont1	G7/cont (Ours)	δSVM/cont (published method)	G3/cont2	G5/cont
F9	**0.6242**	0.3906	0.4889	0.4279	0.5642
GP1BB	0.5559	**0.5661**	0.4206	0.3565	0.3484
HBB	**0.4458**	0.4156	0.3931	0.4394	0.3916
HBG1	0.571	**0.5914**	0.3423	0.459	0.4787
HNF4A	0.3393	**0.3967**	0.1335	0.2936	0.1906
LDLR	**0.5025**	0.4922	0.3399	0.2299	0.351
MSMB	−0.0399	0.1391	**0.1648**	0.0819	0.0628
PKLR	0.6116	**0.6667**	0.4912	0.4927	0.0246
TERT(GBM)	0.5942	**0.6653**	0.4881	0.561	0.5224
TERT(HEK293T)	0.5283	**0.5919**	0.4137	0.4686	0.3609
IRF4	0.3707	**0.527**	0.5023	0.2639	0.0246
IRF6	0.387	**0.444**	0.2641	0.3566	0.2363
MYC	**0.4278**	0.1636	0.3366	0.2847	0.1356
SORT1	0.4743	**0.4982**	0.4471	0.3497	0.2363
ZFAND3	0.3872	0.2145	**0.477**	0.4029	0.2223
Average	**0.451993**	0.45086	0.380213	0.364553	0.276687

### 3.7 Post submission analysis on CAGI5 training data

**Figure 7:**
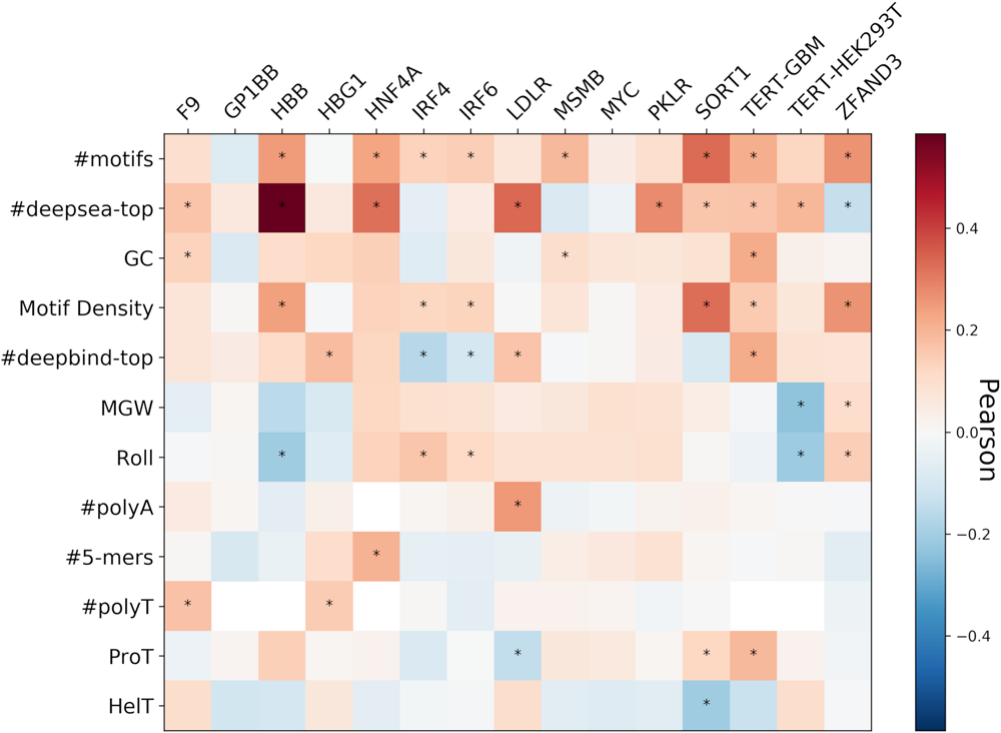
Pearson correlation between variant effect and the difference between variant and wild‐type feature across the relevant individual features for the nine promoters and five enhancers tested in CAGI5. *Cells whose correlation's *p* < 0.05

We examined the correlation between the continuous variant impact and the difference between variant and wild‐type *Sequence features* (Figure 7 and Table S8). We observe that some of the strongest features are the ones highly correlated with MPRA activity as found in previous data sets (Figure 1). We conclude that predictive feature differences for variant impact are consistent with predictive features for MPRA activity.

Following the assessors recommendations (Shigaki et al., 2019), we focused the rest of our analysis on the *continuous challenge*, as the *discrete challenge* did not provide enough data for more in‐depth analysis. The reason for this is that several (3 out of the 14 elements) of the mutagenesis experiments contained as few as three variants exhibiting what was deemed by the organizers as significantly positive or negative impact.

**Figure 8:**
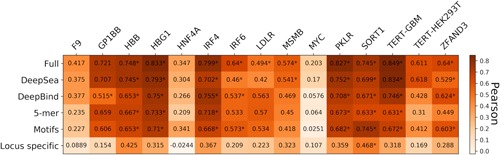
Performance of the regression model with different feature combinations, using Pearson's correlation for nine promoters and five enhancers tested in CAGI5. *Next to correlations with *p *< 0.05

We retrospectively discovered that training one regression model per reference genomic region (14 altogether) to predict variant impact outperforms training one regression model per type (promoter or enhancer), so we proceeded with the former strategy. We observed that the full set of *Sequence features* resulted in the best performance in all of the enhancer and promoter elements, compared with specific feature sets (Figure 8 and Table S9). We do not observe any significant differences between cell‐types in terms of model performance (e.g., changes in activity of perturbing the DNA element telomerase reverse transcriptase [TERT] in HEK293T cells can be predicted with high performance [0.61 Pearson] whereas MYC tested in the same cell line has lower performance ([0.2 Pearson]).

**Figure 9:**
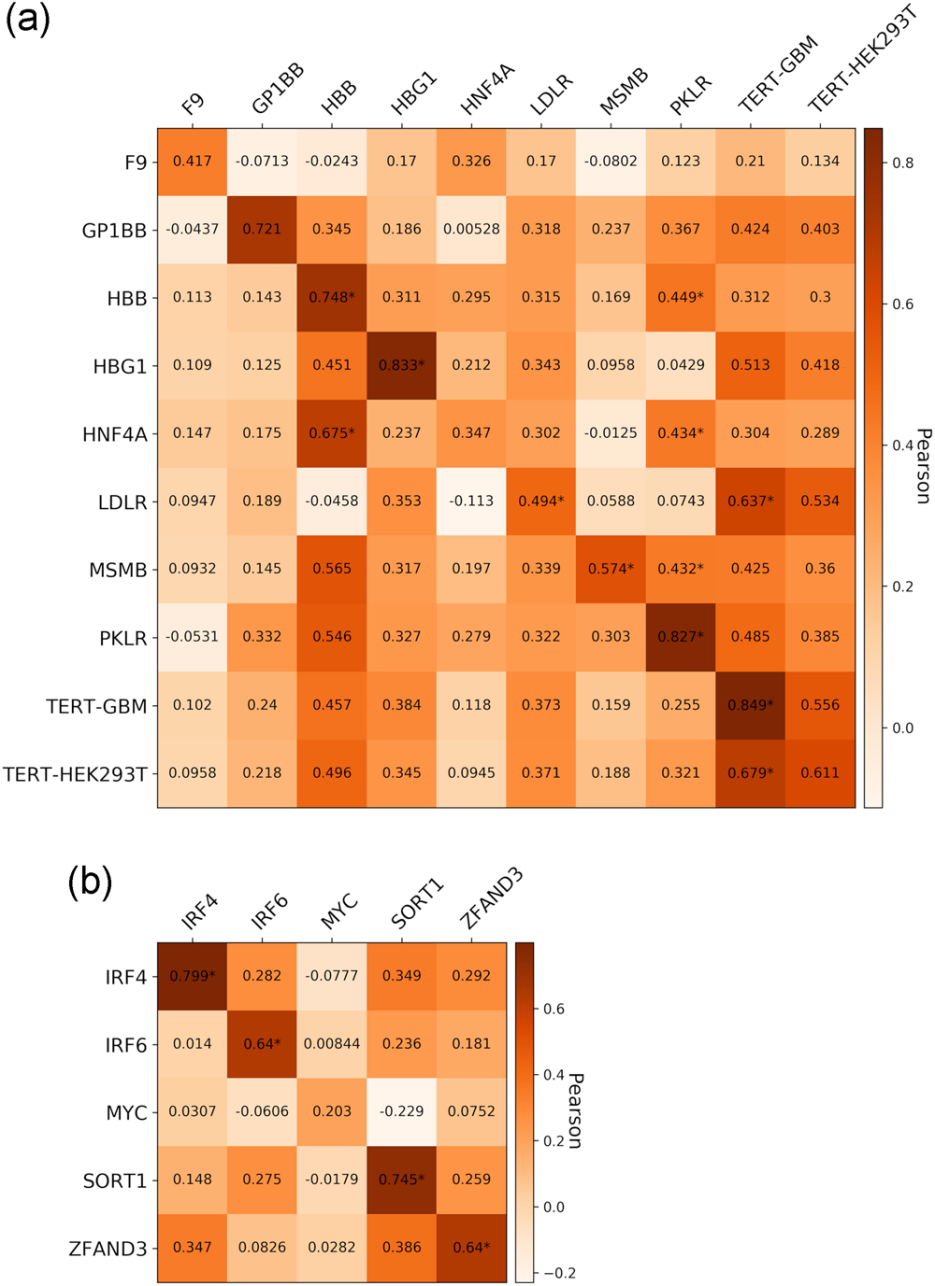
Performance of cross‐data set learning of the regression task for CAGI5 data (a) nine promoters (b) five enhancers. All cross‐data set learning models are ensemble models with full features. Each cell is colored according to the correlation coefficient of the Pearson test. *Next to correlations with *p *< 0.05

To examine whether predictions of SNV effect can be generalized from one data set (here, perturbations of a single DNA element) to the next, we applied the same procedure as before—namely training a regression model on one data set and testing it on the remaining ones (keeping promoters and distal elements separate). We found that the accuracy of the predictions from cross‐data set models were mostly weaker than the predictions from the supervised models (Figure 9 and Table S10). We note that cross‐data set prediction between TERT‐GBM and TERT‐HEK293T, which was trained on the same region but tested in different cell‐types, exhibited moderate strength compared with the supervised predictions. We observe poor performance for cross‐element predictions in the same cell‐type, for example, training on LDLR and testing on F9 (both promoters tested in HepG2 cells) or training on GP1BB and testing on HBG1 (both promoters tested in HEL 92.1.7 cells). These results suggest that for saturation mutagenesis data, supervised prediction models using both the same element and same cell‐type work generally better than cross‐data sets models, highlighting the specificity of local variation presented in this data. However, more data sets are required to substantiate this claim. Generally, the absolute performance for predicting variant effects is substantially lower than that achieved in the task of predicting the transcription of individual sequences, which can be expected as this task relates to a much more nuanced signal.

## 4 DISCUSSION

MPRA holds a great promise to be a key functional tool that will increase our understanding of gene regulatory elements and the consequences of nucleotide changes on their activity. Although previous studies already used MPRA to construct predictive models of transcriptional regulation, its generalizability across cellular contexts and its applicability for studying the endogenous genome have not yet been systematically evaluated. Here, we study MPRA data from a number of cellular systems to determine which features are reflective of the cellular context (e.g., protein milieu in the cell), and which are intrinsic to DNA sequence. We aimed to incorporate the most recently produced MPRA data sets of endogenous sequences in this study, but had to exclude several data sets after quality control analysis (e.g., The data in Maricque, Dougherty, & Cohen (2017)) consisted of few barcodes per candidate enhancer and had significant inconsistency across replicates. The experimental design in Ulirsch et al. (2016) included three genomic regions per enhancer, in overlapping windows. Activity measurements were highly variable between windows of the same enhancer, while many of the features we use were shared among the overlapping windows. We explore the extent by which knowledge of regulatory activity in one cellular context can be used to make predictions in a held out cellular context. Finally, we examine the ability of our framework to detect the effects of small variants on MPRA activity. Our results represent, to the best of our knowledge, the first such comprehensive analysis.

Our work highlights genome accessibility and TF binding as the strongest predictors of regulatory activity, with no observed advantage to cell‐type‐specific features. When applying prediction models, we observe that performance is improved when using an ensemble of all features, with no significant prediction improvement when using cell‐type‐specific features. These results imply that part of the signal observed in MPRA studies is not cell‐type specific. Interestingly, models trained with chromosomal MPRA data yield better predictions across data sets than those trained on episomal MPRA data, stressing the importance of this experimental approach that conveys a more reliable representation of the endogenous settings.

When training on one cell‐type and predicting on another cell‐type, we observe overall lower but robust results, with regions enriched in cell‐type‐specific signal being harder to predict. Notably, we detect a communal component across data sets with a group of TFs being top predictors, as well as some cell‐specific factors that seem to be involved in phenotypes associated with the corresponding cell‐type. In the MPRA setting the *cis* environment (e.g., chromatin) is altered, thus generally not cell‐type specific, and the *trans* environment (e.g., TF binding) remains similar, hence we can still observe predictive factors that are cell‐type specific.

As seen through its performance in the CAGI5 Regulation Saturation challenge, our approach is competitive in the high‐resolution task of predicting the functional effects of SNVs in a supervised setting.

Our work provides a comprehensive resource of annotation for thousands of endogenous sequences across the genome. Furthermore, we demonstrate the performance of different machine learning models for MPRA activity prediction on features generated by publicly available tools. Our approach can highlight functionally important regulatory regions across the genome in a cell‐type agnostic fashion and can be leveraged for an efficient design of future MPRA experiments by prioritizing regions of interest.

## Supporting information

Supporting information

Supporting information

Supporting information

Supporting information

Supporting information

Supporting information

Supporting information

Supporting information

Supporting information

Supporting information

Supporting information
