# Supplementary material for: Meta‐analysis of massively parallel reporter assays enables prediction of regulatory function across cell types
Source: Hum Mutat. 2019 Jun 18;40(9):1299–313. doi: 10.1002/humu.23820 (PMC6771677; doi:10.1002/humu.23820)
Supplement: Supplementary file 1 — Supporting information [file HUMU-40-1299-s001.pdf]

Supplementary Information for "Meta-analysis of massively parallel reporter assays enables prediction of regulatory activity"

<u>Supplementary Figures and Legends</u>

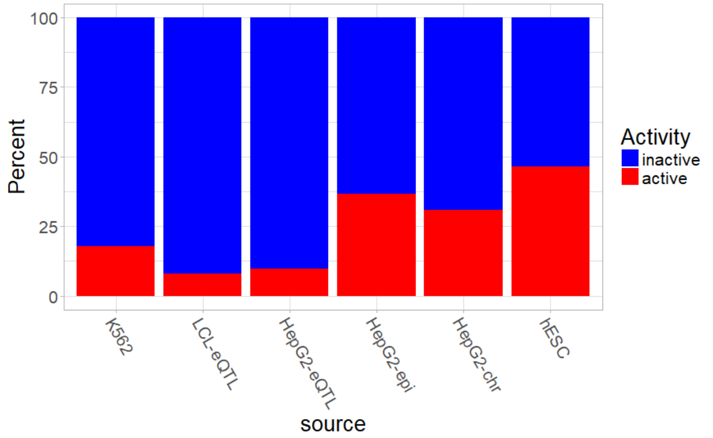

<u>Figure S1:</u> Activity rates of candidate enhancers. Activity was based on MPRAnalyze classification analysis, using MAD-scores (median-based variant of Z-score) of transcription rate estimates, and the distribution of the negative controls as the baseline. Active enhancers are those with FDR < 0.05. The rates of enhancers classified as active are *K562*: 373 / 2100 (17.7%); *LCL-eQTL*: 6258 / 78542 (8%); *HepG2-eQTL*: 7562 / 78719 (9.6%); *HepG2-epi*: 859 / 2338 (36.7%); *HepG2-chr*: 722 / 2338 (30.9%); *hESC*: 1146/2464 (46.5%).

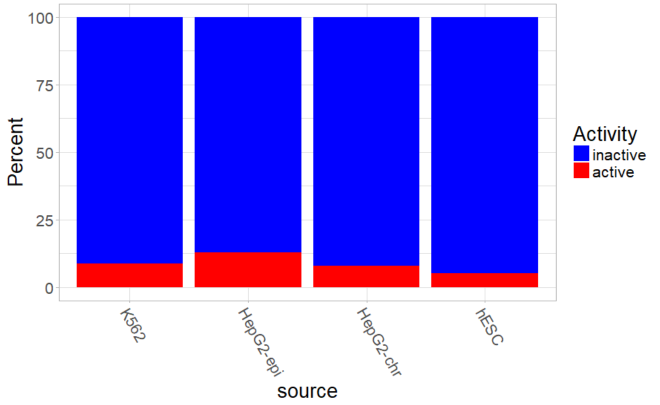

<u>Figure S2:</u> Activity rates of negative control enhancers. Activity was based on MPRAnalyze classification analysis, using MAD-scores (median-based variant of Z-score) of transcription rate estimates, and the distribution of the negative controls as the baseline. Active enhancers are those with FDR < 0.05. The rates of control enhancers classified as active are *K562*: 98 / 1136 (8.6%); *HepG2-epi*: 13/102 (12.7%); *HepG2-chr*: 8 / 102 (7.8%); *hESC*: 10/200 (5%).

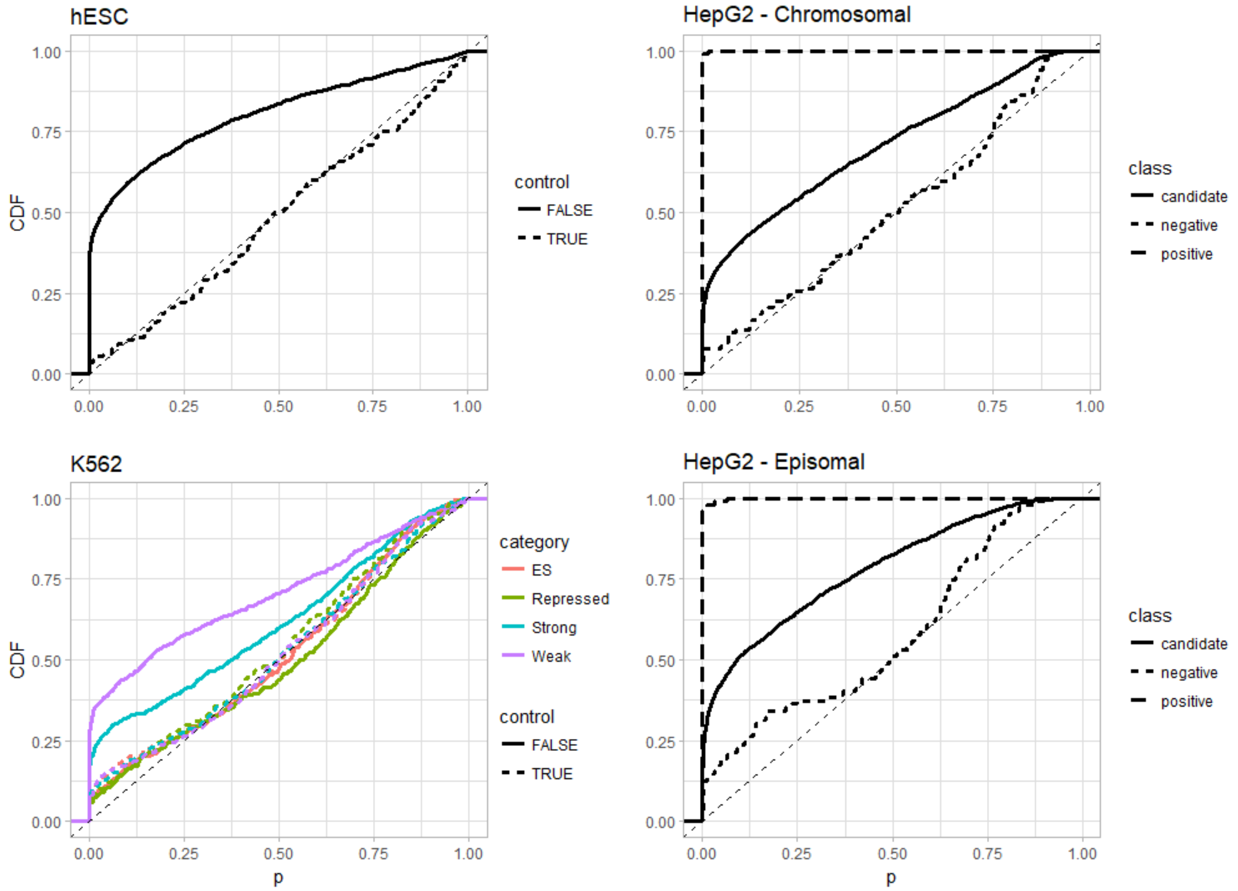

Figure S3: Distribution of p-values computed by the MPRAnalyze classification of active enhancers in each dataset. Closer to left-top corner means higher rate of significant enhancers. Enhancers are divided to positive controls (dashed lines, only available in the HepG2 dataset) candidate enhancers (solid lines) and negative controls (dotted line). Theoretical p-value distribution of inactive enhancers is represented as a thin dotted line. p-values are computed from the MPRAnalyze estimates of transcriptional rates, using the MAD-score (a median-based variant of the Z-score) distribution of the negative controls to establish the baseline other enhancers are tested against.

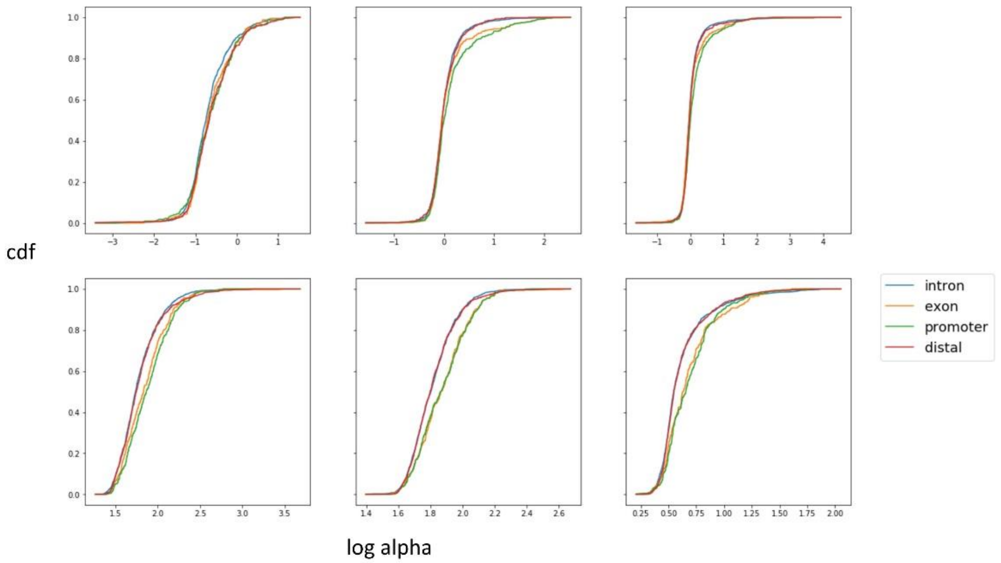

Figure S4: The effect of genomic location (exon, intron, promoter, distal) on MPRA activity per dataset.

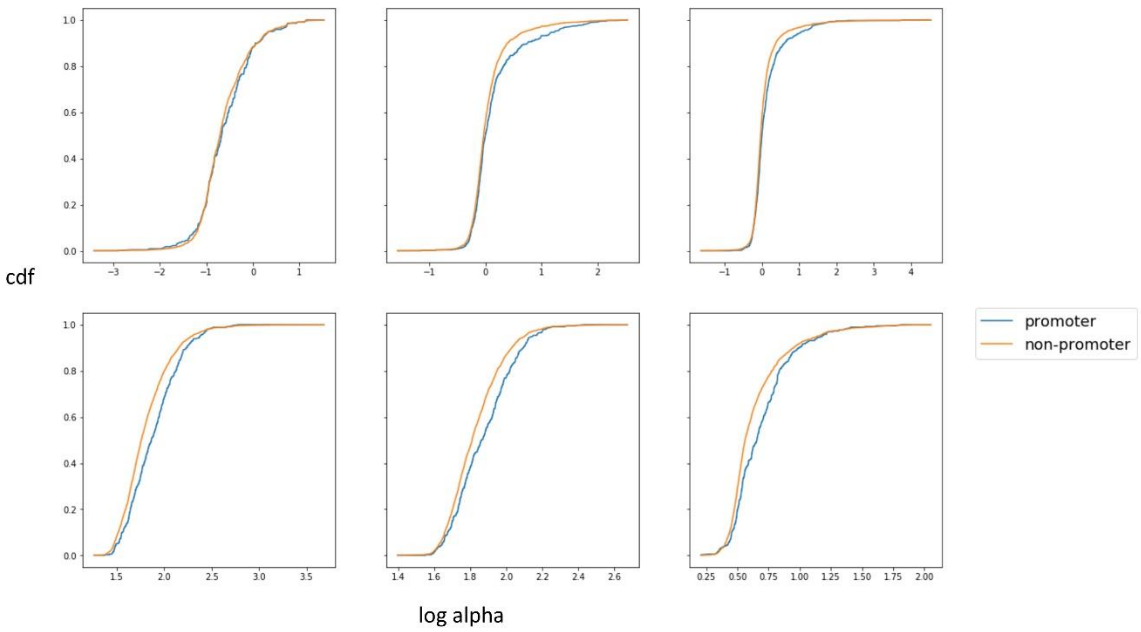

Figure S5: The effect of genomic location (promoter vs. non-promoter) on MPRA activity per dataset.

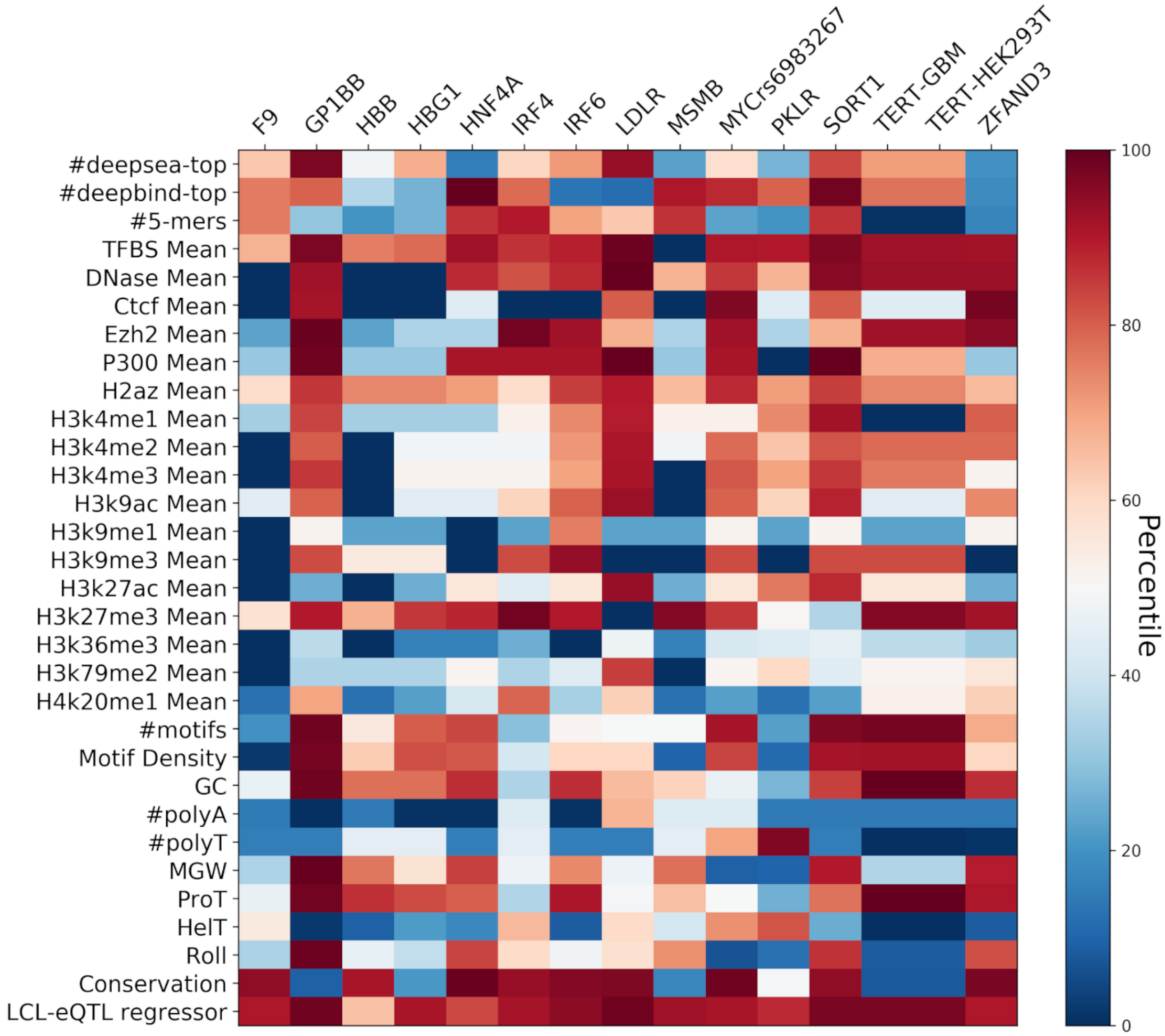

Figure S6: Comparison of single features across 14 regulatory elements from CAGI5 data (centered with a 150bp window) to the distribution of the same features across regions in LCL-eQTL dataset and each cell reports the percentile per feature (white=50% percentile, blue<%50, red>50%). “LCL-eQTL trained regressor” refers to a regression model trained on LCL-eQTL reference regions with the full set of features used to predict the MPRA activity of CAGI5 regions and compared with LCL-eQTL regions activity distribution.

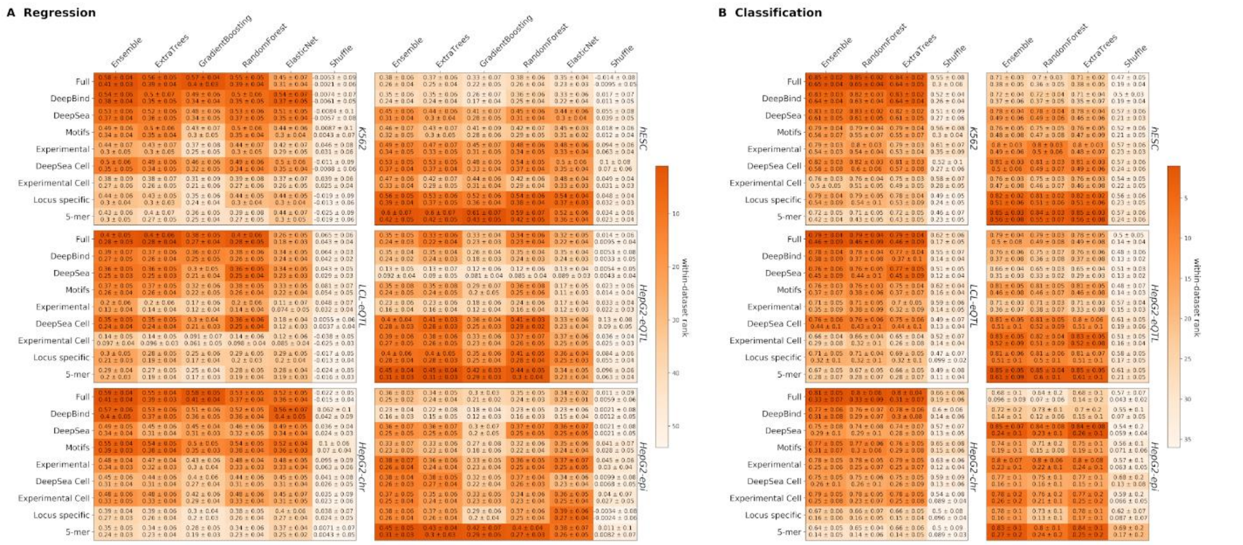

Figure S7: Performance of (A) regression models and (B) classification models with different feature combinations. Note that cell-type specific DeepSea and Experimental features are included, although DeepSea does not have hESC specific TFs. The within-dataset ranking is calculated for each cell by taking the median of the rankings for all the (A) regression or (B) classification tests within a dataset. Each heatmap is colored according to the within-dataset rankings. The statistics are *mean ± std* for (A) Spearman and Kendall tests or (B) AUROC and AUPRC tests.

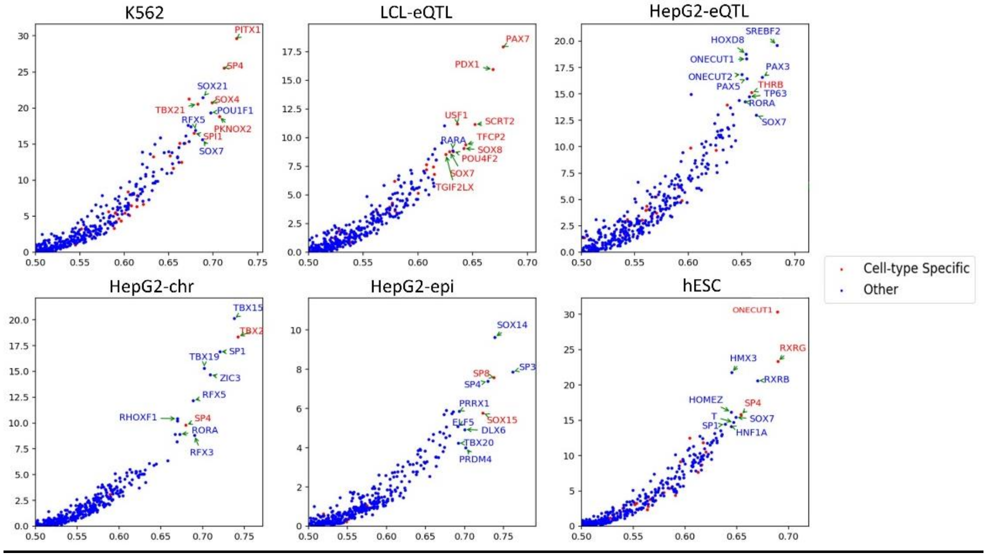

Figure S8: Contribution of individual DeepBind TFs for each dataset, AUROC of the classification versus the –log10 q-value of the classification for the TFs with AUROC greater than or equal to 0.5. Top 10 TFs (by AUROC) are labeled. Red points indicate TFs that are trained on data from the same cell-type as the dataset.

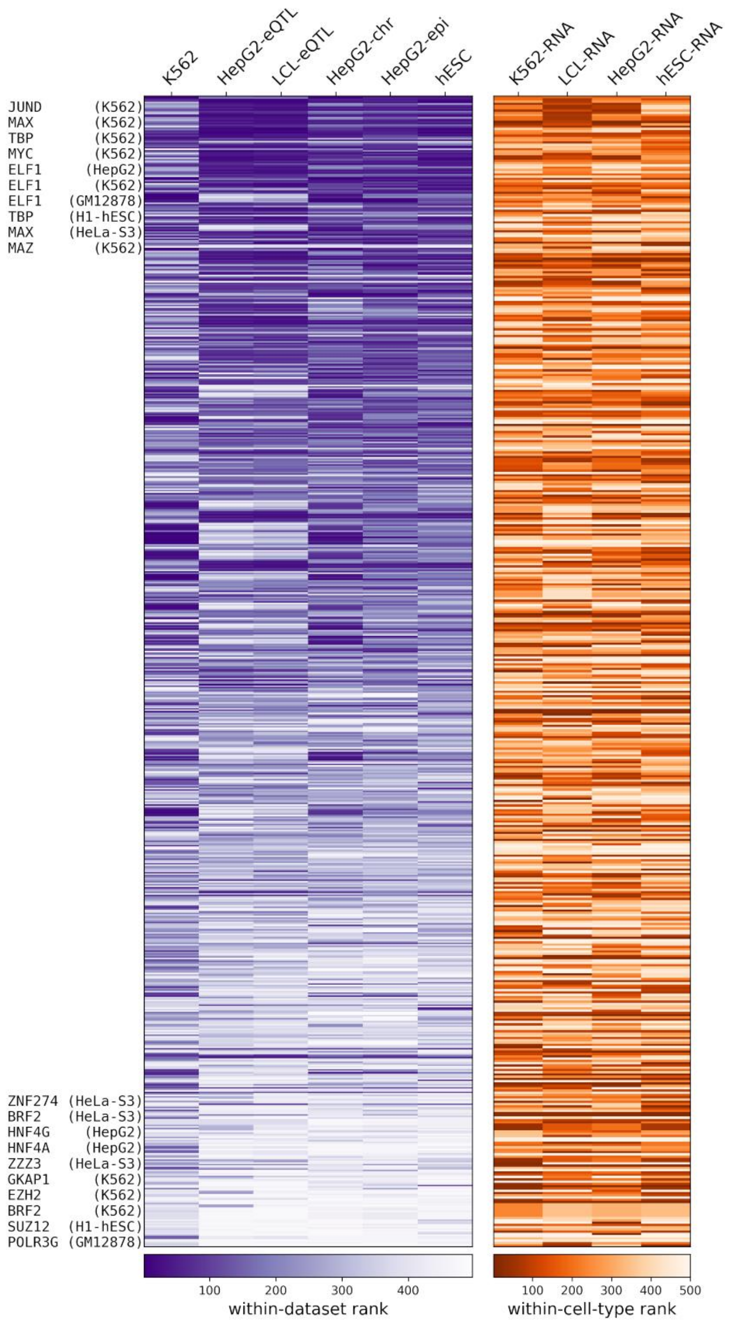

Figure S9: Contribution of individual Experimental TF binding for predicting regulatory activity of MPRA constructs. The within-dataset ranking is calculated by taking the per feature median rank across all classification and regression tests. The comprehensive ranking is the per feature median over all within-dataset rankings. TFs are sorted from best (smallest) to worst comprehensive rank. (left) Heatmap of the within-dataset rankings. (right) the per TF ranking of gene expression measured by RNA-seq in each of the four cell lines. Names of the common top/bottom 10 factor (experimental cell-type) pairs are indicated on the left.

Supplementary Tables legends

Table S1: Single and model features description and single feature predictivity results per dataset.

Table S2: Prediction models results per dataset.

Table S3: Prediction models results per dataset including cell type specific features.

Table S4: Cross- dataset learning results.

Table S5: TF correlations (ChIP/DeepBind) with MPRA activity.

Table S6: TF comparison between datasets.

Table S7: Performance comparison across submissions for the CAGI5 Regulation Saturation *discrete challenge*, sorted by average Pearson correlation.

Table S8: Correlations between variant effect and the difference between variant and wild-type feature across the relevant individual features for the 9 promoters and 5 enhancers tested in CAGI5.

Table S9: Performance of the regression model with different feature combinations, for 9 promoters and 5 enhancers tested in CAGI5.

Table S10: Performance of cross- dataset learning of the regression task for 9 promoters and 5 enhancers tested in CAGI5. All cross- dataset learning models are ensemble models with *sequence features*.
